# Priming in response to pro-inflammatory cytokines is a feature of adult synovial but not dermal fibroblasts

**DOI:** 10.1186/s13075-017-1248-6

**Published:** 2017-02-10

**Authors:** Thomas Crowley, John D. O’Neil, Holly Adams, Andrew M. Thomas, Andrew Filer, Christopher D. Buckley, Andrew R. Clark

**Affiliations:** 1 Rheumatology Research Group, Institute of Inflammation and Ageing, College of Medical and Dental Sciences, University of Birmingham, Queen Elizabeth Hospital, Birmingham, B15 2WB UK; 2 Royal Orthopaedic Hospital NHS Foundation Trust, Bristol Road South, Northfield, Birmingham, B31 2AP UK

**Keywords:** Fibroblast, Rheumatoid arthritis, Stromal memory, Cytokine, Interleukin-6, NF-κB

## Abstract

**Background:**

It has been hypothesized that chronic inflammatory diseases such as rheumatoid arthritis (RA) may be caused by a failure of negative feedback mechanisms. This study sought to examine negative feedback mechanisms in fibroblast-like synoviocytes (FLS), one of the most abundant cell types in the joint. We hypothesized that prior exposure of healthy FLS to an inflammatory stimulus would attenuate their responses to a second inflammatory stimulus, in the same way that negative feedback mechanisms desensitize macrophages to repeated stimulation by lipopolysaccharide. We further hypothesized that such negative feedback mechanisms would be defective in FLS derived from the joints in RA.

**Methods:**

Synovial fibroblasts and dermal fibroblasts from non-inflamed joints and joints affected by RA and a fibroblast cell line from neonatal foreskin were stimulated twice with tumour necrosis factor (TNF) α or interleukin (IL)-1α, with a 24-h rest period between the two 24-h stimulations. Differences between response to the first and second dose of cytokine were examined by assessing secretion of inflammatory factors and intracellular signalling activity.

**Results:**

FLS from both non-inflamed joints and joints affected by RA mounted an augmented response to re-stimulation. This response was site-specific, as primary dermal fibroblasts did not alter their response between doses. The fibroblast priming was also gene-specific and transient. Assessment of signalling events and nuclear localization showed prolonged activation of nuclear factor (NF)-κB during the second stimulation.

**Conclusion:**

This study aimed to examine mechanisms of negative regulation of inflammatory responses in FLS. Instead, we found a pro-inflammatory stromal memory in FLS obtained from both non-inflamed joints and joints affected by RA. This suggests the joint is an area at high risk of chronic inflammation, and may provide a piece in the puzzle of how chronic inflammation is established in RA.

**Electronic supplementary material:**

The online version of this article (doi:10.1186/s13075-017-1248-6) contains supplementary material, which is available to authorized users.

## Background

Rheumatoid arthritis (RA), a chronic inflammatory disease of the joint, is one of the most common autoimmune diseases in the UK, affecting over 1% of the population [1]. In RA, high levels of pro-inflammatory cytokines are maintained within the joint, chemokine gradients continue to recruit leukocytes and the cells within the synovium are retained and are prevented from undergoing apoptosis. Further, the tissue-resident stromal cells become hyperplastic, leading to the growth of an invasive pannus, which narrows the joint space and degrades both articular cartilage and bone.

One of the predominant cell types of the synovial stroma is the fibroblast-like synoviocyte (FLS). It has become increasingly apparent that the FLS is crucial to normal and aberrant inflammatory responses in the joint [2–4]. FLS can release large quantities of pro-inflammatory cytokines. IL-6, for example, is secreted at higher basal levels in FLS in RA than in osteoarthritis (OA) [5]. FLS in RA increase leukocyte adhesion to [6] and transmigration through [7] endothelial layers, and the high concentration of CXCL12 produced by FLS in RA restricts the egress of leukocytes from the joint [8]. Further, FLS release of granulocyte macrophage colony stimulating factor (GMCSF) keeps neutrophils alive for twice the normal span in vitro, and this growth factor is produced at higher levels by FLS in RA than in healthy controls [9–11]. FLS in patients with RA have an increased rate of proliferation [12], are more resistant to apoptosis than FLS in their healthy counterparts [13], and also have increased invasive capabilities and matrix metalloproteinase (MMP) production [14]. Finally, the strongest evidence for the pathogenic role of FLS in RA comes from the experiments of Muller-Ladner et al., who showed that FLS in RA could be cultured in vitro and then implanted in mice with severe combined immunodeficiency (SCID) to induce RA-like disease in the absence of immune cells [15]. Thus, FLS in RA are now viewed as “imprinted aggressors” rather than the traditional “passive responders” [16].

It is well-known that inflammation does not passively end, but is actively switched off [17–21]. It is therefore possible that chronic inflammatory diseases such as RA involve failure of mechanisms in the resolution of inflammation. The negative regulation of inflammation has been well-characterised in monocytes/macrophages (reviewed in [22]). Macrophages respond to a variety of inflammatory stimuli, both endogenous, such as TNFα and IL-1, and exogenous, such as lipopolysaccharide (LPS). Whilst these stimuli elicit strong inflammatory responses, they also induce anti-inflammatory pathways that curtail macrophage inflammation [19, 22, 23]. Negative regulation of inflammation occurs at a range of levels, both within and without the cell. Pro-inflammatory stimuli often elicit anti-inflammatory mechanisms to curtail their own action [22]. This is exemplified by the nuclear factor (NF)-κB-IκBα negative feedback loop. Once a stimulatory signal causes degradation of its inhibitor IκBα, NF-κB is free to translocate to the nucleus and facilitate transcription of pro-inflammatory mediator genes. Another gene also induced by NF-κB is NF-κB-inhibitor-α (Nfkbia), which encodes IκBα. Nascent IκBα shuttles NF-κB out of the nucleus to once again be held inactive, thereby curtailing the transcriptional response to stimulus [24, 25].

Despite the abundant literature on negative regulation of inflammation in macrophages, there is a comparative paucity of information on fibroblasts. Evidence from Lee et al. suggests that FLS in RA lack the inherent negative regulation seen in macrophages [26]. Rather, FLS continued to produce pro inflammatory mediators for the duration of incubation with TNFα, even after four days of stimulation. This paper also showed significantly lower expression of negative regulators such as A20-binding inhibitor of NFκB activation 3 (ABIN3), activating transcription factor 3 (ATF3), IL-1 receptor-associated kinase M (IRAK-M), and suppressor of cytokines signalling 3 (SOCS3) in FLS in RA compared to macrophages. The implication is that FLS are deficient in the negative feedback regulation of inflammation in RA. However, no comparison was made with FLS obtained from non-inflamed joints. A similar study showed that gingival fibroblasts (HGF) lack SOCS1, IRAKM and SH2 domain-containing inositol phosphatase 1 (SHIP1) proteins, which are all used in negative regulation of inflammation [27].

We sought to compare the inflammatory responses of FLS obtained from inflamed (RA) and non-inflamed joints, with the aim of elucidating which (if any) mechanisms are used by FLS to limit their inflammatory responses, and to test the hypothesis that such mechanism(s) would be aberrant or absent in FLS in RA. Experimental design has been based upon the model of endotoxin tolerance in macrophages, which has been extensively used to elucidate mechanisms of negative regulation of inflammatory responses in those cells [28–31]. Exposure of macrophages to a single dose of LPS reprogrammes their responses to a second dose, such that expression of pro-inflammatory mediators is attenuated, whilst expression of anti-microbial products is spared [30, 31]. In a similar fashion, we exposed fibroblasts to two doses of TNFα, with an intervening rest period in the absence of the pro-inflammatory cytokine. We then compared the first and second responses. Contradictory to our initial hypothesis, we found FLS to augment their cytokine-induced IL-6 secretion when primed with either TNFα or IL-1α. We found this priming phenomenon to occur in FLS derived from both inflamed and non-inflamed joints, but not fibroblasts from adult skin. The augmented second response was gene-specific and transient. Sustained activation of NF-κB in response to the second stimulation may play a role in fibroblast priming.

## Methods

### Study participants

Patients with RA who were involved in this study were diagnosed according to the 1987 American College of Rheumatology (ACR) criteria [32]. Synovial tissue was collected during joint replacement surgery. Ultrasound-guided synovial biopsies were collected during arthroscopic examination of unexplained joint pain. Where there was no evidence of inflammatory joint pathology, and no diagnosis of RA was subsequently made, these samples were designated as “healthy” (non-inflamed) controls. Dermal fibroblasts were derived from skin samples collected at the time of joint replacement surgery in patients with OA and from patients with RA. Synovial and dermal fibroblasts were cultured as previously described [9]. The study (National Research Ethics Service (NRES) Committee West Midlands - The Black Country Ref. 07/H1204/191) and all participants in this study gave written, informed consent.

### Cells

Fibroblasts were isolated from synovium and skin as previously described [9]. Cells were grown in Roswell Park Memorial Institute (RPMI) medium supplemented with 10% foetal calf serum, 0.81 × minimum essential medium (MEM) non-essential amino acids, 0.81 mM sodium orthopyruvate, 1.62 mM glutamine, 810 U/mL penicillin and 81 μg/mL streptomycin. Cells were used at passages 3–8. A dermal fibroblast line from neonatal foreskin, BJ (ATCC CRL-2522), was purchased from ATCC. Cells were grown in Eagle’s minimum essential medium (EMEM) (ATCC) supplemented with 10% foetal calf serum, 810 U/mL penicillin and 81 μg/mL streptomycin.

### Experimental design

The repeat dose experiments involved seeding cells and allowing them to adhere overnight, before stimulating cells with vehicle (growth medium), TNFα at 10 ng/mL, or IL-1α at 10 ng/mL (both Peprotech) for 24 h. Conditioned medium was removed and cells were washed thoroughly before being rested for one, three or seven days in fresh medium. In some instances, cells were treated with 100 nM MLN4924 or vehicle control (0.1% dimethyl sulfoxide (DMSO)) during the first stimulation with TNFα, or during the one-day rest period. Cells were washed again after the rest period and stimulated with vehicle, TNFα or IL-1α at the same concentration as above. Conditioned medium was removed after 24 h for analysis.

Assessment of NF-κB activity or localization was based on cells seeded into 6-cm dishes for western blots, and eight chamber glass slides for immunofluorescence studies. Cells were left overnight to adhere, and then stimulated with TNFα (10 ng/mL) at the time points described in the appropriate figures.

### Analysis

Enzyme-linked immunosorbent assay (ELISA) was used to measure the secretion of cytokines. IL-6 (cat #88-7066-88), IL-8 (cat #88-8086-88), and CCL5 (cat #BMS-287/2INST) ELISA kits were purchased from E Bioscience. The latter was used according to manufacturer’s instructions, the two former were used with a 1-ng/mL top standard, in order to capture a greater range of responses to various stimuli.

Western blot analysis was conducted to assess internal protein abundance and activation. BJ cells were lysed in radioimmunoprecipitation assay (RIPA) buffer (150 mM sodium chloride, 1% NP-40, 0.5% sodium deoxycholate, 0.1% SDS, 50 mM Tris pH8). Assessment of nuclear and cytosolic protein localization was performed using a hypotonic lysis buffer and centrifugation to isolate the cytosolic fraction, and a nuclear extraction buffer and centrifugation to isolate the nuclear fraction. Gel blotting was performed using 12-well pre-cast gels and membranes from BioRad. The following western blotting antibodies were all from Cell Signaling Technology: α Tubulin, Lamin A/C, phospho-p38 (T180/Y182), RelA, phospho-RelA (S536), phospho-ERK (T202/Y204) and phospho-JNK (T183/Y185). All antibodies were used at 1:1000 dilution in 5% BSA as blocking reagent, except α Tubulin and RelA, which were at 1:2000 dilution in 5% milk.

### Immunofluorescence

Cells on chamber slides were fixed using 4% paraformaldehyde for 20 minutes, washed three times for 5 minutes in PBS, then permeabilized using 0.1% Triton for 2 minutes before repeat washing with PBS. Slides were then blocked in 10% horse serum for 1 h. NF-κB cellular localization was assessed using a RelA antibody (#H0714 Santa Cruz) used at 1:200 dilution in 1% horse serum and incubated overnight in the dark at 4 °C. Slides were then washed three times for 5 minutes and incubated with a goat anti-rabbit IgG fluorescein isothiocyanate (FITC)-conjugated antibody (#4050-02 Southern Biotech) (1:200) in the dark for 1 h at room temperature. Slides were then mounted in 1,4-diazabicyclo[2.2.2]octane (DABCO) for microscopy analysis. Control chambers received no primary antibody, but received secondary antibody at the same concentration at stated above. As an isotype control, FLS received FITC-conjugated rabbit IgG. Cells were imaged on a Zeiss AxioCam ERc5s with supporting Zen blue edition software.

### Statistical analysis

Repeat dose data are represented either as raw values, or as fold changes from response to the first dose, which is normalised to one. Data are presented as mean with SEM error bars. Comparison of the first and second response to stimulus was conducted after normalisation. The Wilcoxon signed rank test was used, and significance is designated as *p* = 0.05 (*) or *p* = 0.01 (**). Comparison of the first and second response at equivalent time points was analysed by the Mann-Whitney *U* test, with *p* = 0.05 (*). Statistical analyses were performed using GraphPad Prism 6.0 (GraphPad Software).

## Results

### Response of primary human fibroblasts to repeated stimulation with TNF

This study employed FLS from non-inflamed joints and from joints affected by both early and late RA. Many of these lines proliferated slowly and reached replicative senescence after a few passages. It was discovered that the rapidly dividing foreskin fibroblast line BJ behaved very similarly to FLS in terms of responses to re-stimulation. BJ cells were used in some experiments requiring large numbers of cells, and key findings were validated using primary FLS.

**Fig. 1:**
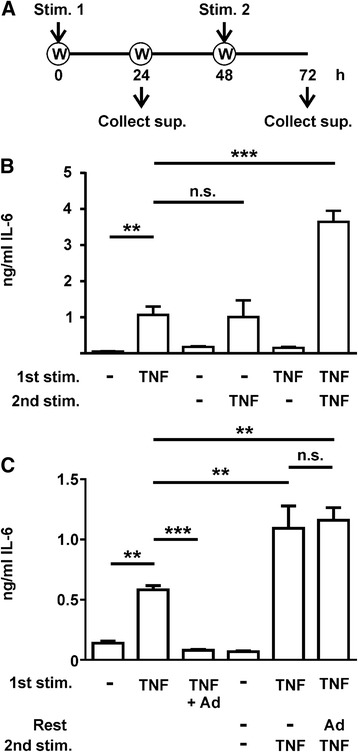
Pre-exposure of neonate dermal fibroblasts to TNF augments their response to re-stimulation. **a** The experimental procedure. BJ fibroblasts received vehicle or 10 ng/mL TNFα for 24 h, and were then washed and rested for 24 h after collection of supernatants. Cells were then washed again and treated with vehicle or 10 ng/mL TNFα for a further 24 h before collection of supernatants (*Collect sup.*). *Stim* stimulation, *W* wash. **b** Fibroblast secretion of IL-6 in response to first or second doses of vehicle or TNFα. **c** Fibroblast secretion of IL-6 under the same conditions, with addition of 1 μg/mL adalimumab (*Ad*) either at the same time as the first TNFα stimulus (*third column*) or during the rest period between the first and second TNFα stimuli (*sixth column*). Mean ± SEM, n = 3–6. **p < 0.01; ***p < 0.005; *n.s.* not statistically significant

BJ cells were used to investigate whether primary human fibroblasts could be tolerized to inflammatory stimuli in the same manner that macrophages become tolerized to LPS [30] (Fig. 1a). Fibroblasts were treated with or without TNF for 24 h, supernatant was collected, cells were washed and rested for a further 24 h, then washed again and treated with or without TNF for a further 24 h. In the absence of stimulus, low amounts of IL-6 were secreted during the first 24 h (Fig. 1b, column 1) or between 48 and 72 h (column 3). If cells were treated with TNF during the first 24 h and then the stimulus was removed, expression of IL-6 was also low between 48 and 72 h (Fig. 1b, column 5), indicating that the continued presence of TNF is necessary for maintaining an inflammatory response. IL-6 expression was induced to a similar extent in cells stimulated at 0 h and harvested at 24 h, or stimulated for the first time at 48 h and harvested at 72 h (Fig. 1b, columns 2 and 4). In contrast, cells that were exposed to repeated TNF stimuli with an intervening 24-h rest period expressed IL-6 at a significantly higher level (Fig. 1b, column 6).

The TNFα-neutralizing antibody adalimumab was used to test whether the augmented second response might be caused by residual signalling of TNFα that had not been effectively removed in the wash step (Fig. 1c): 1 μg/ml adalimumab (*Ad* in Fig. 1) was sufficient to completely block TNFα-induced IL-6 production (compare columns 2 and 3). However, the same concentration of adalimumab during the rest period (24 to 48 h) had no impact on the enhanced response to re-stimulation (compare columns 5 and 6). Prior exposure to TNF therefore primes BJ fibroblasts for an augmented response to re-stimulation with the same cytokine. In subsequent figures, responses to first and second stimulations are compared (equivalent to Fig. 1b columns 2 and 6).

### Priming of fibroblasts in response to pro-inflammatory cytokines is site and gene specific, but not disease or stimulus specific

**Fig. 2:**
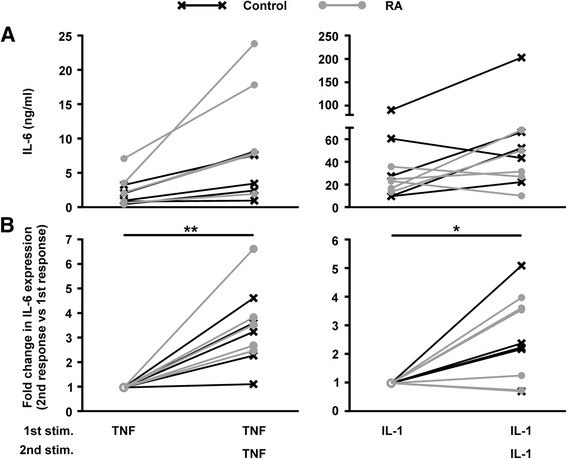
Fibroblast-like synoviocytes (FLS) are primed by pro-inflammatory cytokines whether they are derived from inflamed or non-inflamed joints. FLS were stimulated (*stim*) for 24 h with either TNFα or IL-1α (10 ng/mL) before the stimulus was removed and cells were rested in fresh medium for 24 h. Cells were then washed and stimulated with 10 ng/mL of the same cytokine for a further 24 h. IL-6 secretion in response to the first and second dose was measured by ELISA. **a** Absolute concentrations of IL-6 in supernatants after the first and second exposure to TNFα (*left*) or IL-1α (*right*). **b** IL-6 secretion in response to the second dose, represented as a fold change from the response to the first dose (normalised to 1); n = 10 (5 from non-inflamed joints, 5 from joints affected by rheumatoid arthritis (*RA*)). *Grey circles* and *connecting lines* represent FLS from joints affected by RA. *Black crosses* and *connecting lines* represent FLS from non-inflamed joints. *p < 0.05; **p < 0.01 (Wilcoxon matched pair signed rank test)

RA-derived and non-inflamed control FLS were exposed to repeat doses of TNF or IL-1α with an intervening 24-h rest period as described above, and release of IL-6 in response to the first and second stimulus was measured (Fig. 2). Although there was considerable variability between individual FLS lines in terms of absolute quantities of IL-6 produced, all showed robust increases of IL-6 expression in response to both TNF and IL-1α. Contrary to expectation, there were augmented responses to the second stimulus in almost all cases, and it proved impossible to distinguish between FLS lines on the basis of their origin in RA or non-inflamed synovium. Synovial fibroblast priming in response to pro-inflammatory cytokines, therefore, does not appear to be a disease-specific phenomenon.

**Fig. 3:**
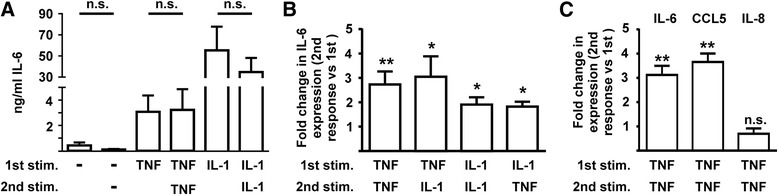
Priming of fibroblasts by pro-inflammatory cytokines is site-specific and gene-specific, but not stimulus-specific. **a** Following the protocol described in Fig. 1, primary human dermal fibroblasts were treated for 24 h with vehicle, TNFα or IL-1α, washed and rested for 24 h, then treated again with vehicle, TNFα or IL-1α for 24 h. Expression of IL-6 protein was measured after the first and second treatments. Mean ± SEM, n = 5. *n.s.* not significant as determined by the Mann-Whitney *U* test. **b** Adapting the protocol described in Fig. 1, BJ fibroblasts were treated for 24 h with TNFα, washed and rested for 24 h then treated with TNFα or IL-1α for 24 h (*left graph*); or treated for 24 h with IL-1α, washed and rested for 24 h, then treated with IL-1α or TNFα (*right graph*). IL-6 expression in response to the second stimulus is represented relative to the response to the same first stimulus. Mean fold change in IL-6 expression between the second and first response ± SEM, n = 6. *p < 0.05 (Wilcoxon matched pair signed rank test). **c** Fibroblast-like synoviocytes (FLS) were treated with TNFα for 24 h, washed and rested for 24 h, then re-stimulated with TNFα. Secreted IL-6, CCL5 and IL-8 were measured after the first and second stimulations, and fold changes in expression calculated. Mean fold change ± SEM, n = 5–8. **p < 0.01; *n.s.* not statistically significant (Wilcoxon matched pair signed rank test)

To test whether fibroblasts from a different anatomical site also demonstrated priming in response to pro-inflammatory stimuli, human dermal fibroblasts (HDFs) were repeatedly stimulated with TNF or IL-1α as in Fig. 1. It should be noted that HDFs were derived from the skin of adults undergoing joint replacement, and behave very differently from the BJ neonatal foreskin fibroblasts investigated in Fig. 1. Expression of IL-6 was induced by TNF or IL-1α in all HDF lines tested (Fig. 3a). Responses to the first and second stimulus were not significantly different. Unlike FLS or BJ cells, dermal fibroblasts are not primed by prior exposure to pro-inflammatory cytokines.

Priming was not stimulus-specific (Fig. 3b). BJ fibroblasts stimulated for 24 h with TNF and then rested for 24 h had augmented responses to re-stimulation with either TNF or IL-1. Likewise, IL-1 effectively primed fibroblasts to respond more strongly to re-challenge with either IL-1 or TNF. The gene specificity of fibroblast priming was next investigated by measuring expression of IL-6, IL-8 and CCL5 in response to the first and second stimulation of FLS with TNF. Like IL-6, the T cell chemokine CCL5 displayed an augmented response to re-challenge with TNF (Fig. 3c), whereas expression of IL-8, a neutrophil chemokine, did not significantly differ in response to the first and second stimulation. This gene specificity indicated that FLS priming was unlikely to be explained trivially by increased cell number during the rest period, or by receptor sensitization.

### Fibroblast priming is transient, and accompanied by changes in cytokine-induced intracellular signaling

**Fig. 4:**
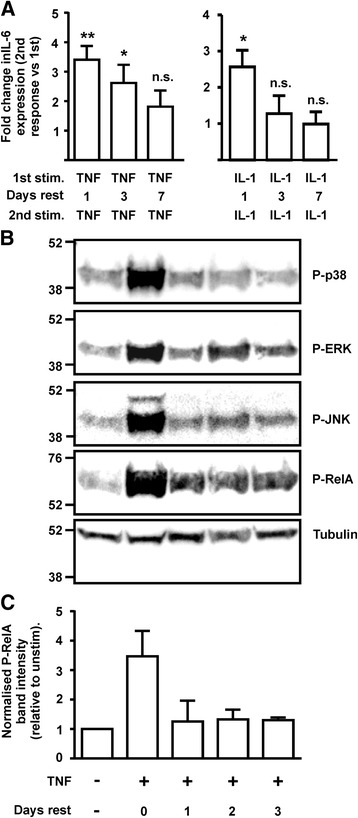
Fibroblast priming is transient. **a** Fibroblast-like synovioctyes (FLS) were stimulated (*stim*) with TNFα or IL-1α (both 10 ng/mL) for 24 h, washed and rested for one, three or seven days, then washed and stimulated again for 24 h. Responses to the first and second dose were analysed by IL-6 ELISA, and response to the second dose was represented as a fold change from response to first dose (normalised to one). Mean ± SEM, n = 5–10. *p < 0.05, **p < 0.01 (Wilcoxon matched pairs signed rank test); *n.s*. not significant **b** BJ fibroblasts were treated with vehicle or TNFα for 24 h then washed and rested in fresh medium for one, two or three days. Whole cell lysates were prepared, and phosphorylated forms of signalling molecules were detected by western blotting. **c** Phospho-RelA was quantified in three independent experiments, normalized against the loading control tubulin and plotted relative to the level in unstimulated cells

To investigate the duration of fibroblast priming by pro-inflammatory cytokine, we conducted the repeat dose experiment on FLS but lengthened the rest period between stimulations (Fig. 4a). The FLS used in these experiments were derived from both RA and non-inflamed joints, which did not significantly differ in their actions (not shown). There was inverse correlation between the length of the rest period and the magnitude of the second response. The priming effect of TNF (fold difference between the second and first response) was 3.41 after one day of rest (*p* = 0.002), 2.62 after three days of rest (*p* = 0.031) and 1.81 after seven days of rest (not significant). The priming effect of IL-1 also declined from one day of rest, becoming statistically insignificant thereafter. Therefore pro-inflammatory cytokine priming does not induce a permanent change in the responsiveness of FLS.

**Fig. 5:**
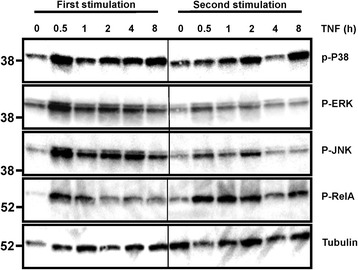
Fibroblast priming is accompanied by changes in signalling responses to TNF. BJ fibroblasts were treated for varying times with TNFα (10 ng/mL) with or without a prior 24 h exposure to TNFα and 24 h rest. Cell lysates were prepared and phosphorylated signalling proteins were detected by western blotting. Results are representative of five independent experiments. *Vertical lines* separate sections from the same gel and exposure. *ERK* extracellular signal-related kinase, *JNK* c-Jun N-terminal kinase

The activation of NF-κB and mitogen-activated protein kinase (MAPK) signalling pathways by pro-inflammatory cytokines was relatively prolonged in BJ cells, remaining significantly above basal levels even 24 h after addition of stimulus (Fig. 4b, c). It was therefore speculated that priming might reflect residual activity of pro-inflammatory signalling pathways after withdrawal of the stimulus, gradually declining during the rest interval. However, activation of NF-κB, extracellular signal-related kinase (ERK), c-Jun N-terminal kinase (JNK) and MAPK p38 all declined to basal levels within 24 h of withdrawal of the cytokine stimulus (Fig. 4b, c). There was no difference in resting NF-κB or MAPK activity between cells rested for one day or for three days after withdrawal of TNF. The responses of the same signalling pathways to the first and second stimulation were then assessed. The activation of MAPKs in response to a second challenge with TNF was consistently attenuated (Fig. 5), an effect that was particularly evident in the cases of ERK and JNK. The first stimulation with TNF induced transient phosphorylation of the NF-κB subunit RelA at serine 536, a post-translational modification associated with NF-κB activation. In contrast the phosphorylation of Ser 536 in response to a second TNF challenge was prolonged.

**Fig. 6:**
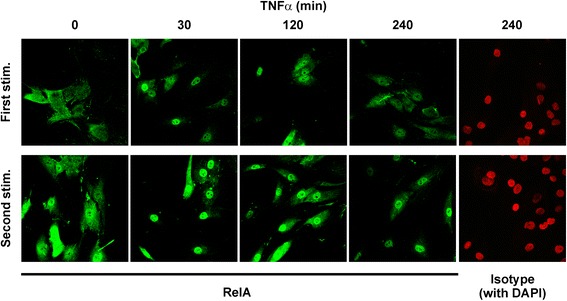
Re-stimulation of fibroblast-like synoviocytes (FLS) with TNFα induces prolonged nuclear localization of RelA. FLS were seeded onto chamber slides and stimulated (*stim*) with 10 ng/mL TNFα for the times indicated (*upper images*), or stimulated with TNFα for 24 h, washed and rested for 24 h, then re-stimulated for the times indicated (*lower images)*. RelA was detected by immunfluorescence microscopy. The *far right* images in each row indicate isotype controls for the primary antibody, with 4′,6-diamidino-2-phenylindole (*DAPI*) staining of nuclei shown in *red*. Combined RelA and DAPI images are shown in Additional file 1: Figure S1. Images are representative of four experiments using different FLS lines from rheumatoid arthritis

The duration of TNFα-induced NF-κB signaling was further investigated by immunofluorescence microscopy in RA FLS. RelA was mostly cytoplasmic prior to the first stimulus (Fig. 6, top left). Discrete nuclear staining was observed 30 and 120 minutes after addition of TNFα, but by 240 minutes RelA was more broadly distributed or localized to the cytoplasm. Prior to the second stimulus, strong staining for RelA was observed in the nuclei of a few cells (Fig. 6, bottom left). Distinct nuclear staining was seen in the majority of cells at 30, 120 minutes and 240 minutes. Corresponding images with nuclear 4′,6-diamidino-2-phenylindole (DAPI) stain are shown in Additional file 1: Figure S1.

**Fig. 7:**
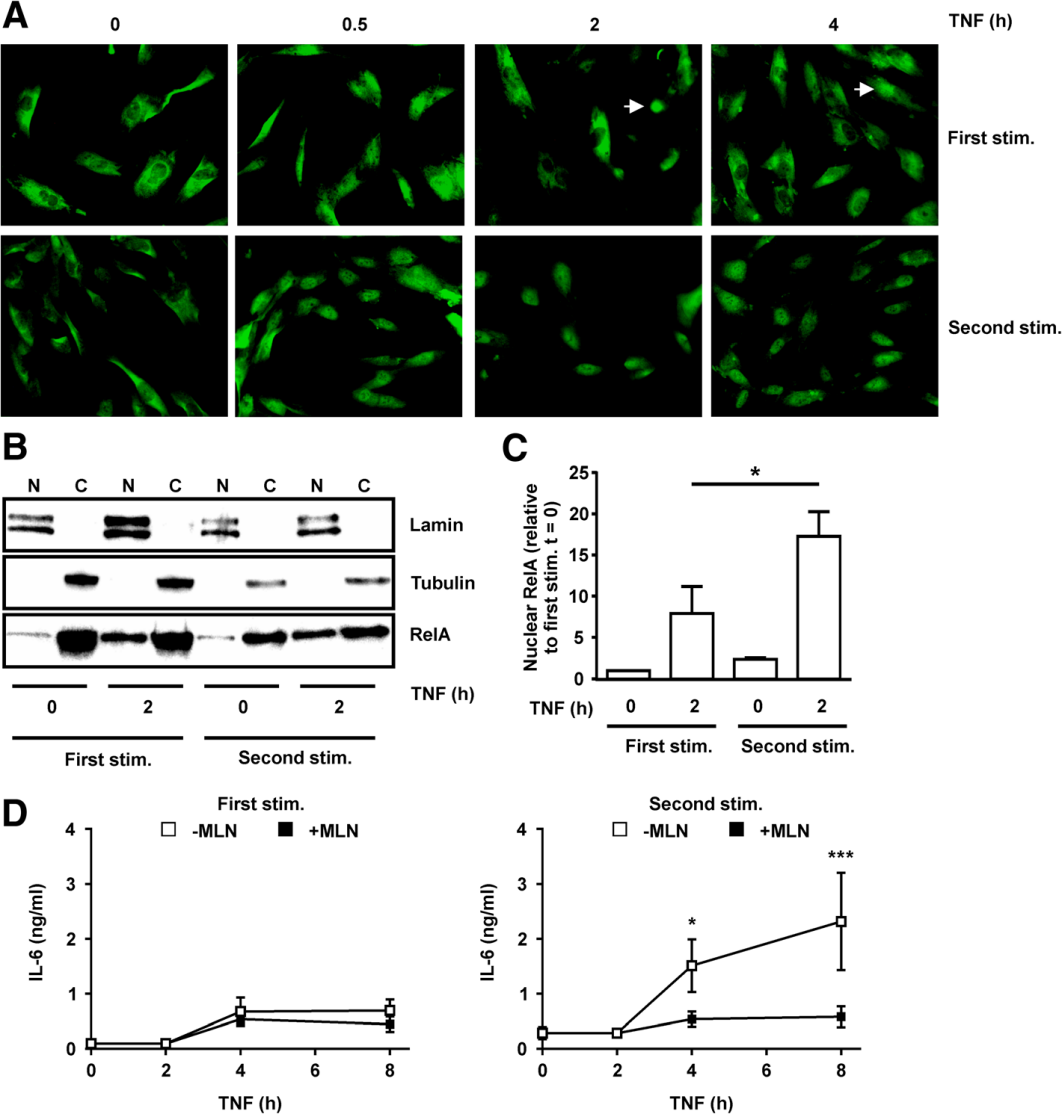
Prolonged nuclear factor (NF)-κB activation in re-stimulated BJ cells contributes to enhanced IL-6 biosynthesis. **a** BJ fibroblasts were seeded on chamber slides and stimulated with TNFα (10 ng/mL) for varying times, with or without prior exposure to TNFα for 24 h and rest in the absence of TNFα for 24 h. RelA was detected by immunofluourescence. Images are representative of two independent experiments. *White arrows* indicate cells in which RelA is principally nuclear. **b** BJ fibroblasts were untreated or stimulated (*stim*) with TNFα (10 ng/mL) for 2 h, with or without priming as above. Nuclear (*N*) and cytoplasmic (*C*) fractions were prepared and RelA was detected by western blotting. Lamin A/C and tubulin were blotted in order to validate the subcellular fractions. Results are representative of four independent experiments. **c** Nuclear RelA was quantified, normalised against the Lamin A/C loading control, and plotted relative to the level at t = 0 in the first TNFα challenge. Mean ± SEM, n = 4; *p < 0.05 (Mann-Whitney *U* test). **d** BJ fibroblasts were stimulated (*left*) or re-stimulated (*right*) with 10 ng/mL of TNFα, and 100 nM MLN4924 or vehicle control (0.1% dimethyl sulfoxide (DMSO)) was added after 2 h. Supernatants were collected at the time points indicated, and IL-6 measured by ELISA; n = 6; *p < 0.05, ***p < 0.005; Student *t* test

Similarly prolonged accumulation of nuclear RelA in response to re-stimulation of BJ cells was confirmed by immunofluorescence microscopy (Fig. 7a) and cell fractionation (Fig. 7b, c). To investigate the significance of prolonged NF-κB activation, BJ cells were stimulated or re-stimulated with TNFα, and the NF-κB inhibitor MLN4924 [33] or vehicle control was added after 2 h (Fig. 7d). IL-6 biosynthesis in response to the first TNFα stimulus did not increase strongly between 2 h and 8 h, and was not significantly impaired by addition of MLN4924 at 2 h. In contrast, IL-6 biosynthesis in response to the second TNFα stimulus increased markedly between 2 h and 8 h, and this increase was prevented by MLN4924. These data suggest that prolonged NF-κB signalling beyond the 2-h time point contributes to the enhanced expression of IL-6 after re-stimulation.

## Discussion

LPS tolerance involves a range of epigenetic and signalling mechanisms that extensively reprogramme the responses of macrophages to re-stimulation, suppressing the induction of overtly pro-inflammatory genes whilst sparing those with anti-microbial properties [28–31]. This may be viewed as a form of innate immune memory that guards against excessive inflammation without compromising defence against pathogens. The present study was devised to identify equivalent negative regulatory mechanisms by which synovial fibroblasts constrain their responses to repeated pro-inflammatory challenge. Surprisingly, we instead found that synovial fibroblasts were primed by a first exposure to either TNFα or IL-1α, such that they secreted significantly greater amounts of IL-6 when challenged a second time, after a 24-h rest period.

Fibroblasts derived from RA synovium retain an imprinted, invasive phenotype throughout prolonged culture in vitro [15, 16, 34]. Nevertheless, we found no evidence that the priming phenomenon was specific to RA-derived synovial fibroblasts. Similar priming responses were demonstrated by synovial fibroblasts from ostensibly healthy, non-inflamed joints of patients who did not have RA, and who did not later develop RA. A neonatal foreskin fibroblast line was primed by TNFα or IL-1α in a manner very similar to synovial fibroblasts, and was used in this study as a tool to explore mechanisms. In contrast, adult dermal fibroblasts (obtained from patients with OA and patients with RA at the time of joint surgery) showed no evidence of priming.

Synovial fibroblasts from joints affected by RA were previously shown to mount unremitting inflammatory responses, continuing to express IL-6 and other inflammatory mediators for at least four days in the presence of TNFα [26]. The same group also demonstrated priming by TNFα of synovial fibroblasts in RA, resulting in enhanced responses to later challenge with interferons [35]. In those studies no comparison was made with “normal” synovial fibroblasts. It is therefore possible that the properties described are characteristic of synovial fibroblasts as a whole, rather than acquired during the pathogenesis of RA. It is not a novel concept that fibroblasts differ in their patterns of gene expression and response to stimulation, according to the anatomical location from which they are obtained [11, 36–38]. The important questions are why synovial fibroblasts should possess “inflammatory memory” [35], and whether such memory could play a role in the development of chronic joint disease.

One argument is that fibroblast responses to pro-inflammatory stimuli are dictated by their usual pattern of exposure to such stimuli [27]. In mucosal or dermal fibroblasts that are repeatedly exposed to pro-inflammatory insults, priming responses would be severely maladaptive. In contrast, fibroblasts from privileged sites such as the joint or the eye presumably seldom face sustained or repeated exposure to pro-inflammatory cytokines in healthy individuals, and may have evolved different responses to such danger signals. This hypothesis will be tested by investigating priming responses of fibroblasts from different anatomical locations of healthy and diseased individuals. Another possibility is suggested by the apparently gene-specific nature of the phenomenon, in which primed responses of IL-6 and CCL5 were enhanced, whereas that of IL-8 was not. Perhaps, as an inflammatory response evolves, differential priming allows the stroma to influence leukocyte recruitment and activation by modifying the profile of cytokines and chemokines expressed. This possibility is also under investigation.

Finally, the molecular basis of synovial fibroblast priming remains unknown. Increased expression of cytokine receptors is unlikely to provide an explanation, because of the gene-specific and signaling-pathway-specific effects described. Sustained activation of NF-κB has been implicated in the unremitting inflammatory response of synovial fibroblasts to prolonged TNFα exposure in RA [26]. Although we found NF-κB activity to decline after withdrawal of TNFα, the dynamics of activation in response to the second challenge were altered, with sustained nuclear localization of the RelA subunit and phosphorylation of its serine 536. It appears that the first exposure of FLS to TNFα induces a transient burst of *IL6* gene expression, whereas the second exposure induces more sustained expression, dependent on prolonged activation of NF-κB. We speculate that priming influences the expression or function of negative feedback regulators required for the termination of the NF-κB activation signal.

## Conclusion

We demonstrated here that inflammatory memory is a general property of synovial fibroblasts rather than a peculiarity of synovial fibroblasts in RA. We show that this phenomenon is transient, gene-specific rather than universal, and is accompanied by alterations of cytokine-induced signal transduction pathways. We hypothesize that the propensity of synovial fibroblasts to respond more strongly to re-stimulation contributes to the development of chronic joint inflammation.

## Additional file

Additional file 1: Figure S1. Re-stimulation of FLS with TNFα induces prolonged nuclear localization of RelA. FLS were seeded onto chamber slides and stimulated with 10 ng/mL TNFα for the times indicated (*upper images*), or stimulated with TNFα for 24 h, washed and rested for 24 h, then re-stimulated for the times indicated (*lower images*). Cells were stained with DAPI to identify nuclei, and RelA was detected by immunofluorescence microscopy. The same images without DAPI stain are shown in Fig. 6 (TIF 11558 kb)

## Abbreviations

ABIN: A20 binding inhibitor of NFκB activation
ATF: Activating transcription factor
BSA: bovine serum albumin
DAPI: 4′,6-diamidino-2-phenylindole
DMSO: dimethyl sulfoxide
ELISA: enzyme-linked immunosorbent assay
ERK: extracellular signal-related kinase
FLS: Fibroblast-like synoviocyte
GMCSF: Granulocyte/macrophage colony stimulating factor
HDF: Human dermal fibroblast
HGF: Human gingival fibroblast
IL: Interleukin
IRAK: Interleukin-1 receptor-associated kinase
JNK: c-Jun N-terminal kinase
LPS: Lipopolysaccharide
MAPK: Mitogen-activated protein kinase
MMP: Matrix metalloproteinase
NF: nuclear factor
OA: Osteoarthritis
PBS: phosphate-buffered saline
RA: Rheumatoid arthritis
SCID: Severe combined immunodeficiency
SHIP: SH2 Domain-containing inositol phosphatase
SOCS: Suppressor of cytokine signalling
STAT: Signal transducer and activator of transcription
TNF: Tumour necrosis factor
