# Supplementary figures and images for: Priming in response to pro-inflammatory cytokines is a feature of adult synovial but not dermal fibroblasts
Source: Arthritis Res Ther. 2017 Feb 10;19:35. doi: 10.1186/s13075-017-1248-6 (PMC5303242; doi:10.1186/s13075-017-1248-6)

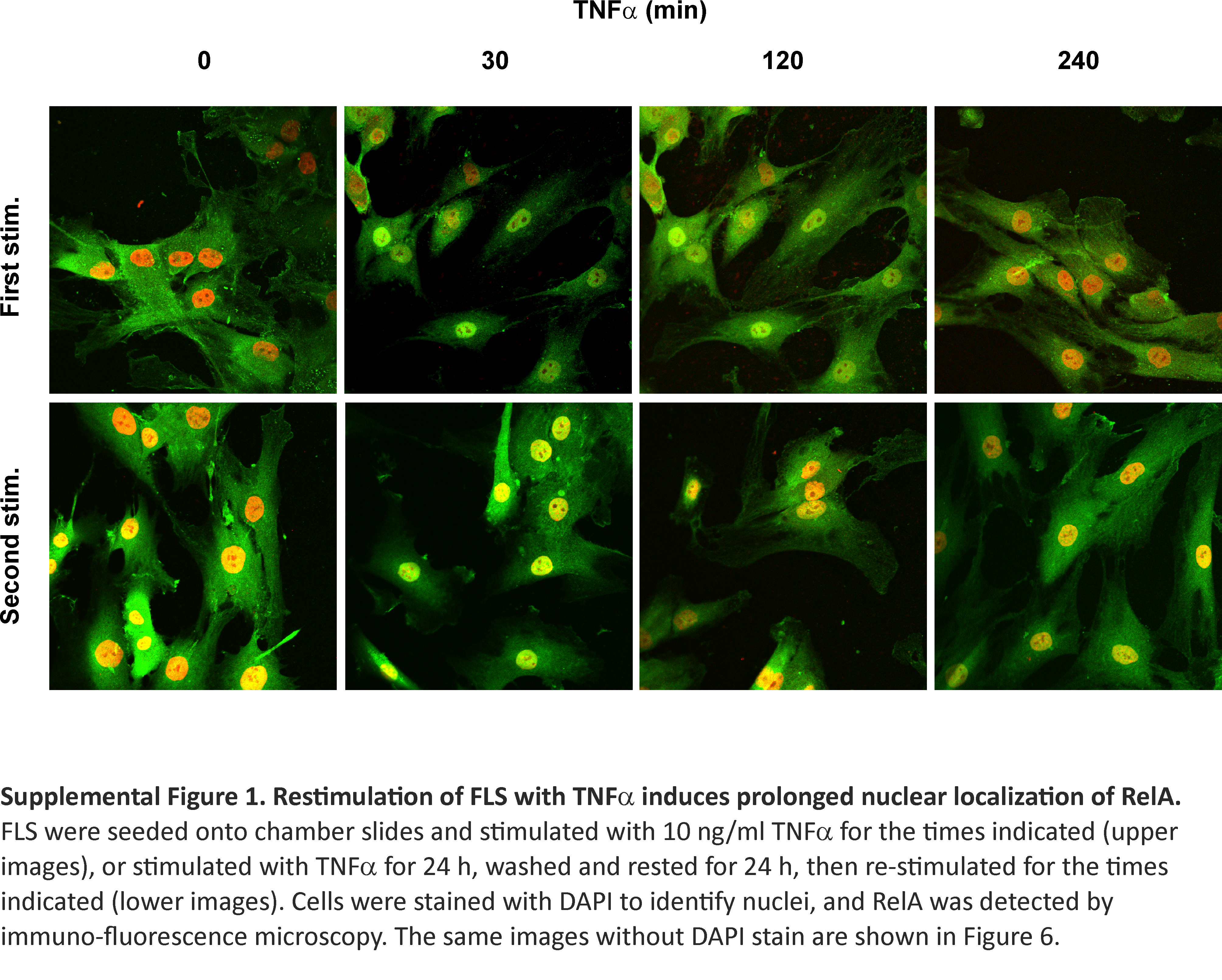

Supplement: Additional file 1: Figure S1. — Re-stimulation of FLS with TNFα induces prolonged nuclear localization of RelA. FLS were seeded onto chamber slides and stimulated with 10 ng/mL TNFα for the times indicated (upper images), or stimulated with TNFα for 24 h, washed and rested for 24 h, then re-stimulated for the times indicated (lower images). Cells were stained with DAPI to identify nuclei, and RelA was detected by immunofluorescence microscopy. The same images without DAPI stain are shown in Fig. 6 (TIF 11558 kb) [file 13075_2017_1248_MOESM1_ESM.tif]
